# Growth factor receptors IGF-1R and VEGFR2 are associated with the prognosis of patients with esophageal cancer after esophagectomy

**DOI:** 10.1515/biol-2025-1354

**Published:** 2026-07-27

**Authors:** Tiehong Zhang

**Affiliations:** Department of Oncology, Shandong Provincial Hospital Affiliated to Shandong First Medical University, Jinan, Shandong, 250021, China

**Keywords:** esophageal cancer, growth factor receptors, prognosis, nomogram

## Abstract

This single-center retrospective study evaluated the prognostic value of IGF-1R, VEGFR2, PDGFR, and c-MET expression in 94 patients with pathologically confirmed ESCC who underwent esophagectomy, and explored the potential relevance of combining immune checkpoint inhibitors (ICIs) with growth factor receptor tyrosine kinase inhibitors (TKIs). A prognostic nomogram was developed for clinically available factors and growth factor receptors with significant prognostic value, and the nomogram was validated with ROC curve analyses. Among the four growth factor receptors, VEGFR2 and IGF-1R expression differed significantly between death and survival groups. Worse prognosis was associated with advanced clinical stage, deeper tumor invasion, greater nodal involvement, high VEGFR2 expression, and high IGF-1R expression (clinical stage: HR = 2.15, 95 % CI: 1.45–3.20; T stage: HR = 1.78, 95 % CI: 1.12–2.83; N stage: HR = 2.42, 95 % CI: 1.61–3.64; VEGFR2: HR = 1.61, 95 % CI: 1.04–2.49; IGF-1R: HR = 1.74, 95 % CI: 1.12–2.70). PDGFR and c-MET expression were not significantly associated with cancer-specific survival. Although the nomogram showed prognostic performance (AUC = 0.512), the small sample size precluded formal histology-specific stratified analysis, limiting assessment of subtype-specific prognostic effects.

## 1 Introduction

Esophageal cancer is the sixth most common cause of cancer death worldwide [1], 2].

There are two major histological types of esophageal cancer, esophageal squamous cell carcinoma (ESCC) and esophageal adenocarcinoma (EAC). In China, however, esophageal cancer is predominantly ESCC, whereas EAC is uncommon [3].

ESCC is characterized by aggressive behavior, early metastatic potential, and a dismal prognosis, with an estimated five-year survival rate below 20 % [4], 5]. Even among patients undergoing curative-intent esophagectomy, clinical outcomes vary considerably, and clinicopathological staging alone does not fully capture individualized cancer-specific risk. Consequently, identifying prognostic biomarkers that complement established clinical factors remains an important unmet clinical need [4], 5].

Angiogenesis, the development of new blood vessels, plays a key role in tumor growth, invasion, and metastasis [6], 7]. Vascular endothelial growth factor (VEGF) promotes vascular permeability as well as endothelial cell proliferation and migration [8], and its receptor VEGFR2 is a major mediator of VEGF signaling and a therapeutic target being investigated in esophageal cancer [9]. Platelet-derived growth factor (PDGF) contributes to angiogenesis by recruiting perivascular cells and stabilizing new vessels [10], and dysregulation of PDGFR signaling has been associated with tumor proliferation and metastasis [11]. The insulin-like growth factor-1 receptor (IGF-1R), a receptor tyrosine kinase, regulates proliferation and survival and has been implicated in ESCC biology [12]. In addition, c-MET (the receptor for hepatocyte growth factor) promotes tumor growth, angiogenesis, and epithelial–mesenchymal transition and has been associated with aggressive disease behavior in esophageal cancer [13], 14]. Together, these receptors form key growth factor/RTK axes that may influence prognosis through angiogenesis, proliferation, invasion, and metastatic potential [6], [7], [8], [9], [10], [11], [12], [13], [14].

However, the comparative prognostic value of VEGFR2, IGF-1R, PDGFR, and c-MET – when assessed together in the same surgically treated esophageal cancer cohort and integrated into an internally validated prognostic tool – remains poorly defined. In addition, limited subtype-aware reporting has made it unclear whether prognostic associations differ between ESCC and EAC [3], [4], [5], [6], [7], [8], [9], [10], [11], [12], [13], [14].

In this study, we assessed the expression and prognostic significance of four growth factor receptors, IGF-1R, VEGFR2, PDGFR, and c-MET, in 94 patients with ESCC who underwent esophagectomy. We further developed a clinicomolecular nomogram to estimate cancer-specific survival probabilities at clinically relevant time points, aiming to support individualized postoperative risk stratification.

## 2 Materials and methods

### 2.1 Patients

This single-center retrospective study included 94 patients with pathologically confirmed ESCC who underwent esophagectomy. Given the limited sample size, a formal power calculation indicated that the study had approximately 70 % power to detect a hazard ratio of 2.0 for cancer-specific survival at a significance level of 0.05.

Inclusion criteria: (1) histologically confirmed esophageal cancer; (2) curative-intent esophagectomy with **R0** resection; (3) availability of adequate formalin-fixed paraffin-embedded (FFPE) tumour tissue for immunohistochemistry; (4) availability of key clinicopathologic variables required for analysis; (5) follow-up information sufficient to determine cancer-specific survival (CSS).

Exclusion criteria: (1) non-curative resection (**R1/R2**) or palliative surgery; (2) multiple primary malignancies; (3) perioperative mortality (death during index hospitalization or within 30 days after surgery); (4) serious postoperative complications precluding outcome evaluation; (5) inadequate or uninterpretable tumour tissue/IHC staining; (6) loss to follow-up preventing outcome ascertainment.

Tumour grade and ESCC diagnosis were confirmed by pathological examination of surgical specimens. No patients with EAC were included in this cohort. Neoadjuvant therapy status was assessed from medical records; no patients received preoperative (neoadjuvant) anti-cancer therapy prior to surgery (chemotherapy, radiotherapy, immunotherapy, targeted therapy, or anti-angiogenic therapy).

Patients were followed from the date of surgery until cancer-specific death or last contact, with a median follow-up of 36 months (range: 6–60 months). Follow-up duration and event/censoring counts are reported in the Results section.

**Informed consent:** Informed consent was obtained from all individuals included in this study, or their legal guardians or wards.

**Ethical approval:** The research related to human use has been complied with all the relevant national regulations, institutional policies and in accordance with the tenets of the Helsinki Declaration, and has been approved by the Research Ethics Committee of Shandong Provincial Hospital (SZRJJ: NO. 2021-180).

### 2.2 Data collection and immunohistochemistry

Clinical, pathological, and demographic data were obtained from medical records. Collected variables included age, sex, clinical stage, tumour–node–metastasis (TNM) stage, tumour size, tumour differentiation, smoking and alcohol history, postoperative complications, and survival outcomes. Histological subtype was not included as a comparative variable because all patients were diagnosed with ESCC. Data on postoperative adjuvant therapies (e.g., chemotherapy, radiotherapy, immunotherapy, and anti-angiogenic therapy) were also recorded. Adjuvant therapy was defined as any systemic therapy and/or radiotherapy administered after esophagectomy and was analysed as a binary variable (yes/no) and, where available, by modality.

Tumor tissue samples were examined for protein expression of four growth factor receptors (IGF-1R, VEGFR2, PDGFR, and c-MET) using immunohistochemistry (IHC) [15]. Sections (3–4 μm) of FFPE tumor tissue were deparaffinized, rehydrated, and subjected to heat-induced antigen retrieval in 10 mM citrate buffer (pH 6.0). The primary antibodies used were: IGF-1R (clone 24–57, 1:200, Cell Signaling Technology), VEGFR2 (clone 55B11, 1:150, Cell Signaling Technology), PDGFR (clone Y92, 1:100, Abcam), and c-MET (clone SP44, 1:250, Roche). Visualization was performed using an HRP-polymer/EnVision detection system with DAB chromogen. Staining localization was predominantly membranous for VEGFR2 and IGF-1R, and cytoplasmic for PDGFR and c-MET.

Briefly, FFPE tumour specimens were fixed in 10 % neutral-buffered formalin, embedded in paraffin, and sectioned at **3–4 μm**. Sections were deparaffinized and rehydrated, followed by heat-induced antigen retrieval using 10 mM citrate buffer (pH 6.0) for 15 min at 95–98 °C. Endogenous peroxidase activity was blocked using 3 % hydrogen peroxide (H_2_O_2_) for 10 min at room temperature, followed by protein blocking using 5 % bovine serum albumin (BSA) for 30 min at room temperature. Slides were incubated with primary antibodies against IGF-1R, VEGFR2, PDGFR, and c-MET (manufacturer, clone/catalog number, dilution, and incubation conditions are provided in Supplementary Table S1) and then with an appropriate secondary detection system (HRP-polymer/EnVision detection kit), with visualization using 3,3′-diaminobenzidine (DAB) and counterstaining with hematoxylin.

The IHC scoring system used an H-score method, in which staining intensity (graded from 0 to 3) was multiplied by the percentage of positively stained cells to yield a score from 0 to 300. H-score was calculated as: Σ (intensity score × percentage of cells at that intensity), yielding a total score from 0 to 300. Two pathologists independently evaluated slides while blinded to outcomes. Inter-observer agreement was evaluated using Cohen’s kappa (*κ*) statistic, and discrepant scores were resolved by consensus review.

Biomarker expression was dichotomized into “high” and “low” groups using two methods: the median H-score and Youden-index cut-offs derived from ROC analysis for 3-year cancer-specific survival (CSS). Median H-scores were IGF-1R = 160, VEGFR2 = 95, PDGFR = 105, and c-MET = 90. Sensitivity analyses confirmed consistent results with Youden-index thresholds.

In addition to median-based dichotomization, sensitivity analyses were performed using Youden-index cut-offs derived from ROC analysis for 3-year CSS. Biomarker expression was also analysed as a continuous variable using restricted cubic splines (RCS) to reduce information loss from dichotomization.

### 2.3 Study design and definition of outcomes

This was a retrospective observational study designed to evaluate the prognostic value of growth factor receptor expression in surgical patients with esophageal carcinoma. The primary outcome was cancer-specific survival (CSS), defined as the time from esophagectomy to death attributable to esophageal cancer. Patients who were alive at the last follow-up (or who died of other causes, when applicable) were censored on the corresponding date. For descriptive baseline comparisons, patients were categorized according to their status at the last follow-up as either the survival group or the death group.

### 2.4 Statistical analysis

SPSS version 19.0 (SPSS Inc., Chicago, IL, USA) and R software version 4.2 (R Foundation for Statistical Computing, Vienna, Austria) were used to perform statistical analyses. Descriptive statistics were calculated for all clinical and molecular variables. Group comparisons between survivors and non-survivors used chi-square (χ^2^) tests for categorical variables. Survival differences were analysed using the Kaplan–Meier method, and statistical differences between survival curves were determined using the log-rank test.

Variables for multivariate Cox regression were selected based on clinical relevance and univariate analysis (P < 0.1), including clinical stage, T stage, N stage, adjuvant therapy, and receptor expression. Given the small cohort (*n* = 94) and 56 events, overfitting risk was acknowledged. The prognostic nomogram was internally validated using bootstrap resampling (1,000 iterations), ROC analysis, and calibration plots, as ROC alone is insufficient to confirm prognostic utility. The base model included clinical stage, T stage, N stage, and adjuvant therapy (yes/no) as clinically relevant covariates. Histological subtype was not included because all enrolled patients had ESCC. Histological subtype and adjuvant therapy were retained and reported in the final multivariable model table regardless of statistical significance.

Proportional hazards assumptions were assessed using Schoenfeld residuals. Collinearity was assessed using variance inflation factors (VIF). Interactions between biomarker expression and histological subtype were not assessed because all cases were ESCC. To minimize instability due to sparse events in certain categories, clinically adjacent categories were collapsed where appropriate (e.g., Stage I–II vs III–IV; T1–T2 vs T3–T4), and sensitivity analyses were performed to confirm robustness of estimates.

All variables were first assessed for the proportion of missing observations. For categorical and continuous variables with less than 5 % missing data, complete-case analysis was performed, as the impact on statistical power and bias was considered negligible. No variable exceeded the 5 % missingness threshold in the final dataset. Specifically, complete data were available for age, sex, histological subtype, clinical stage, T stage, N stage, tumor differentiation, smoking and alcohol history, adjuvant therapy status, and follow-up outcomes. For the four biomarker H-scores (IGF-1R, VEGFR2, PDGFR, c-MET), there were no missing values because immunostaining and scoring were successfully completed for all 94 included cases. Given the absence of any variable with missingness exceeding 5 %, no multiple imputation was performed. A sensitivity analysis comparing results with and without listwise deletion confirmed the robustness of the findings, as the direction and magnitude of hazard ratios remained essentially unchanged. All analyses reported are therefore based on the complete-case dataset (*n* = 94). Missing data patterns were also examined and showed no evidence of systematic missingness (e.g., no association between missing status and clinical outcomes).

Model performance was evaluated using ROC analysis and Harrell’s concordance index (C-index). Internal validation was undertaken using bootstrap resampling with 1,000 repetitions, and calibration was assessed using calibration plots at 1, 3, and 5 years. Decision curve analysis (DCA) was performed to assess net clinical benefit across threshold probabilities.

A two-tailed P value < 0.05 was considered statistically significant. Terminology was standardized to “higher/lower” biomarker levels and “more/less advanced” stage categories, rather than “increased/reduced,” to ensure consistency.

## 3 Results

### 3.1 Comparison of clinical characteristics of esophageal cancer patients with different survival status

A total of 94 consecutive patients diagnosed with esophageal cancer were included and grouped into two categories: (1) the survival group (*n* = 38) and (2) the death group (*n* = 56). Baseline clinical characteristics, including demographics, tumor features, stage distribution, and biomarker expression according to survival status, are summarized in Table 1. Key differences in categorical variables such as clinical stage, T stage, N stage, VEGFR2, and IGF-1R expression are additionally visualized as bar graphs in Figure S1 a–e to facilitate comparison between survival and death groups.

**Table 1:** Comparison of clinical variables of esophageal cancer patients with different survival statuses.

Variables	Survival group (N = 38)	Death group (N = 56)	χ2	*P*-Value
Sex			0.734	0.392
Women	8 (21.05 %)	8 (14.29 %)		
Men	30 (78.95 %)	48 (85.71 %)		
Age			0.068	0.795
1	20 (52.63 %)	31 (55.36 %)		
2	18 (47.37 %)	25 (44.64 %)		
pTNM stage			16.067	<0.001
Ⅰ	8 (21.05 %)	0 (0.00 %)		
Ⅱ	25 (65.79 %)	33 (58.93 %)		
III	5 (13.16 %)	20 (35.71 %)		
T			13.100	0.004
1	7 (18.42 %)	0 (0.00 %)		
2	11 (28.95 %)	12 (21.43 %)		
3	18 (47.37 %)	39 (69.64 %)		
4	2 (5.26 %)	5 (8.93 %)		
N			13.820	0.001
0	33 (86.84 %)	29 (51.79 %)		
1	4 (10.53 %)	11 (19.64 %)		
2	1 (2.63 %)	16 (28.57 %)		
Smoking			1.004	0.316
No	8 (21.05 %)	17 (30.36 %)		
Yes	30 (78.95 %)	39 (69.64 %)		
Drinking			0.012	0.911
No	14 (36.84 %)	20 (35.71 %)		
Yes	24 (63.16 %)	36 (64.29 %)		
Complications			0.001	0.972
No	33 (86.84 %)	50 (89.29 %)		
Yes	5 (13.16 %)	6 (10.71 %)		
Differentiation			3.079	0.079
Well	4 (10.53 %)	1 (1.79 %)		
Moderate	33 (86.84 %)	52 (92.86 %)		
Poor	1 (2.63 %)	3 (5.36 %)		
PDGFR			2.890	0.089
Low	21 (55.26 %)	21 (37.50 %)		
High	17 (44.74 %)	35 (62.50 %)		
VEGFR2			3.956	0.047
Low	11 (28.95 %)	7 (12.50 %)		
High	27 (71.05 %)	49 (87.50 %)		
c-Met			1.518	0.218
Low	14 (36.84 %)	14 (25.00 %)		
High	24 (63.16 %)	42 (75.00 %)		
IGF-1R			8.157	0.004
Low	18 (47.37 %)	11 (19.64 %)		
High	20 (52.63 %)	45 (80.36 %)		

Comparison of demographic, clinical, and molecular features between survival and death groups in esophageal cancer patients. P-values based on the Chi-square test. T stage: depth of primary tumor invasion (T1, tumor invades lamina propria or submucosa; T2, tumor invades muscularis propria; T3, tumor invades adventitia; T4, tumor invades adjacent structures). N stage: regional lymph node involvement (N0, no regional nodes; N1, 1–2 nodes; N2, ≥3 nodes).

**Follow-up and outcomes:** The median follow-up duration was 36 months (range: 6–60 months). During follow-up, there were 56 cancer-specific deaths (CSS events) and 38 censored observations; censored observations refer to patients who were alive at last contact or lost to follow-up before experiencing the event of interest (cancer-specific death), and thus their survival time was only partially observed.

**Figure 1:**
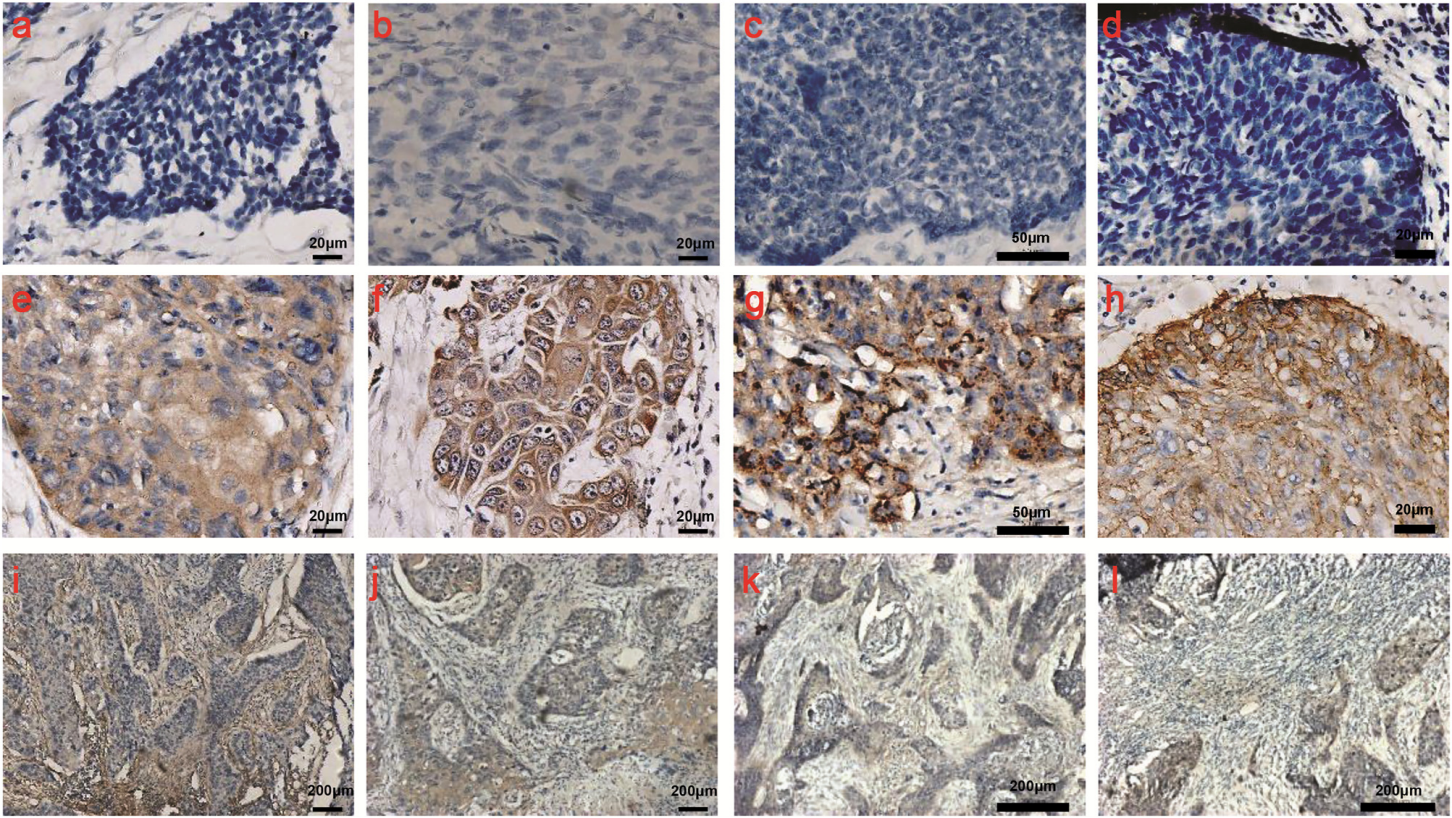
Immunohistochemical expression of PDGFR, VEGFR2, c-MET, and IGF-1R in esophageal cancer tissues. Representative immunohistochemical staining images showing **low** and **high** expression of PDGFR, VEGFR2, c-MET, and IGF-1R (a–h, × 400). Matched low-power images from the same patient are shown (i–l, × 100). Scale bars are included on all micrographs (length indicated on the images).

Representative immunohistochemical staining patterns of IGF-1R, VEGFR2, PDGFR, and c-MET are shown in Figure 1a–h, with matched low-power images from the same patient shown in Figure 1i–l (scale bars are provided on all micrographs; see Figure 1 legend).

When comparing survival status groups, statistically significant differences were observed for clinical stage (P < 0.0001), T stage (P = 0.004), N stage (P = 0.001), high VEGFR2 expression (P = 0.047), and high IGF-1R expression (P = 0.004). No significant differences were observed for PDGFR (P = 0.524) or c-MET (P = 0.372) expression. There were no statistically significant differences in age, sex, smoking status, alcohol use, tumor differentiation between the survival and death groups (Table 1).

### 3.2 Survival analysis

**Figure 2:**
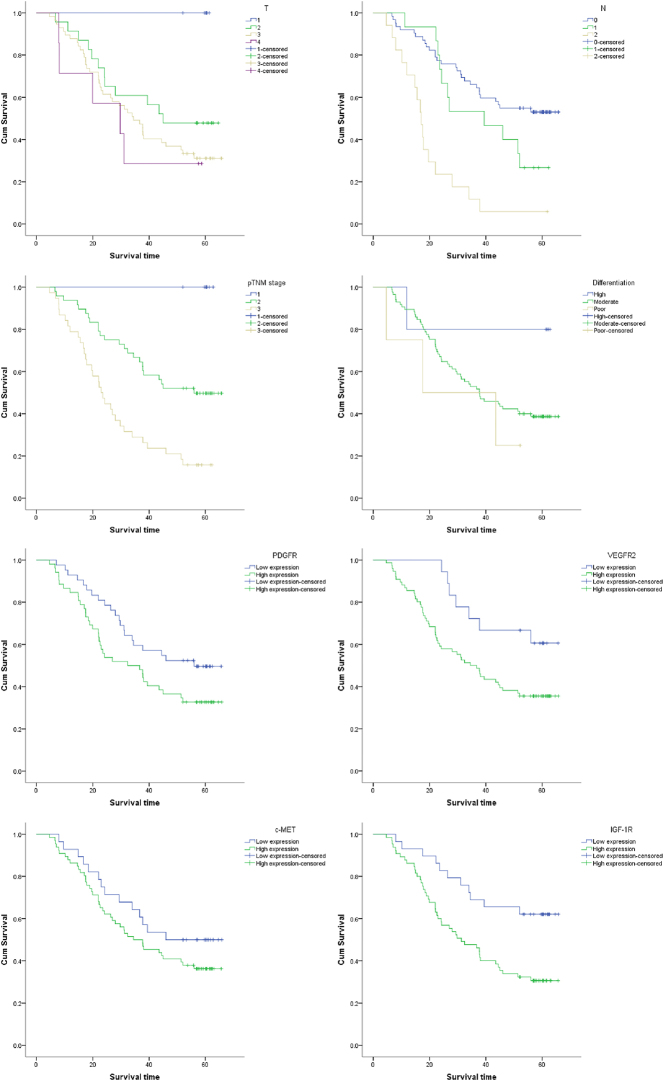
Kaplan–Meier curves of cancer-specific survival stratified by clinicopathological factors and biomarker expression. Kaplan–Meier cancer-specific survival curves according to clinical stage, T stage, N stage, and expression status of VEGFR2, IGF-1R, PDGFR, and c-MET. Group differences were compared using the log-rank test.

Kaplan–Meier survival analysis with log-rank testing was performed to evaluate the association of clinicopathological variables and biomarker expression with CSS. More advanced clinical stage (P < 0.001), more advanced T stage (P = 0.024), more advanced N stage (P < 0.001), high VEGFR2 expression (P = 0.033), and high IGF-1R expression (P = 0.005) were associated with shorter CSS (Table 2). PDGFR (P = 0.588) and c-MET (P = 0.461) were not significantly associated with CSS. Kaplan–Meier curves are presented in Figure 2.

**Table 2:** The survival differences of patients with different clinical features (log–rank test).

Variables	Number	Survival time	χ2	*P*-Value
pTNM stage			16.750	<0.001
Ⅰ	8	–		
Ⅱ	58	–		
Ⅲ	25	22.267 (15.533–37.800)		
T			9.438	0.024
1	7	–		
2	23	–		
3	57	–		
4	7	–		
N			31.859	<0.001
0	62	47.271 (41.902–52.641)		
1	15	39.711 (30.847–48.575)		
2	17	20.251 (13.806–26.696)		
VEGFR2			4.555	0.033
Low	18	53.103 (45.312–60.894)		
High	76	38.575 (33.487–43.662)		
IGF-1R			7.738	0.005
Low	29	51.125 (43.771–58.480)		
High	65	36.978 (31.653–42.303)		

Log-rank test results showing survival time differences among esophageal cancer patients stratified by clinical and molecular features.

The multivariate analysis showed that advanced clinical stage III–IV (HR = 5.394, 95 % CI: 1.357–21.442), certain N stages (N1: HR = 0.044, 95 % CI: 0.009–0.207; N2: HR = 0.234, 95 % CI: 0.078–0.699), high VEGFR2 expression (HR = 2.115, 95 % CI: 0.924–4.839), and high IGF-1R expression (HR = 3.901, 95 % CI: 1.774–8.580) were associated with survival outcomes. T stage and clinical stage II did not reach statistical significance in this cohort, likely due to the limited number of events.

### 3.3 Identifying independent prognostic factors and development of a prognostic nomogram

A multivariable Cox proportional hazards model was constructed to identify independent predictors of CSS. Clinical stage, T stage, N stage, lower VEGFR2 expression, and lower IGF-1R expression remained significant independent prognostic factors after mutual adjustment (Table 3).

**Table 3:** Multivariate Cox regression analysis of risk factors of survival in patients with esophageal cancer.

Variables	B	SE	Wald	P	HR (95%CI)
Clinical stage (vs. Ⅰ)					
Ⅱ	−10.557	368.05	0.001	0.977	–
III	1.685	0.704	5.727	0.017	5.394 (1.357–21.442)
T (vs. 1)					
2	0.743	388.489	0	0.998	–
3	−0.862	0.583	2.191	0.139	0.422 (0.135–1.322)
4	−0.118	0.515	0.053	0.818	0.888 (0.324–2.436)
N (vs. 0)					
1	−3.132	0.796	15.497	<0.001	0.044 (0.009–0.207)
2	−1.452	0.559	6.76	0.009	0.234 (0.078–0.699)
VEGFR2	0.749	0.422	3.144	0.076	2.115 (0.924–4.839)
IGF-1R	1.361	0.402	11.46	0.001	3.901 (1.774–8.580)

Multivariate Cox regression analysis identifying independent risk factors associated with survival in esophageal cancer patients. B, regression coefficient (log hazard ratio); SE, standard error of the coefficient; Wald, Wald chi-square statistic; P, P-value; HR, hazard ratio; 95 %CI, 95 % confidence interval. Reference categories: Clinical stage (vs. I), T stage (vs. I), N stage (vs. 0).

**Figure 3:**
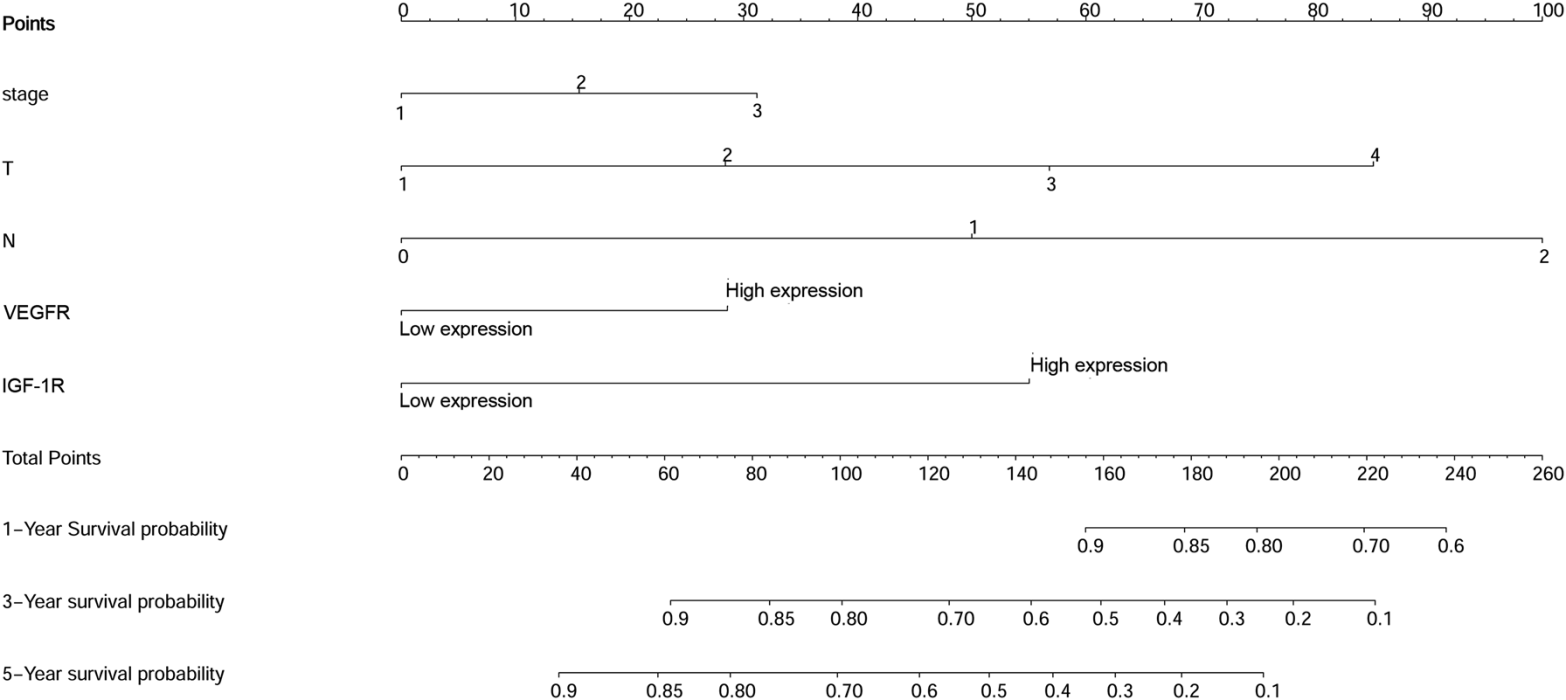
Nomogram for predicting cancer-specific survival after esophagectomy in esophageal cancer patients. Nomogram integrating clinicopathological factors and biomarker expression to estimate individual 1-, 3-, and 5-year cancer-specific survival probabilities. Points assigned to each variable are summed to obtain a total score, which corresponds to the predicted survival probability.

Based on the final multivariable model, a nomogram was developed to estimate individual 1-, 3-, and 5-year CSS probabilities (Figure 3).

**Figure 4:**
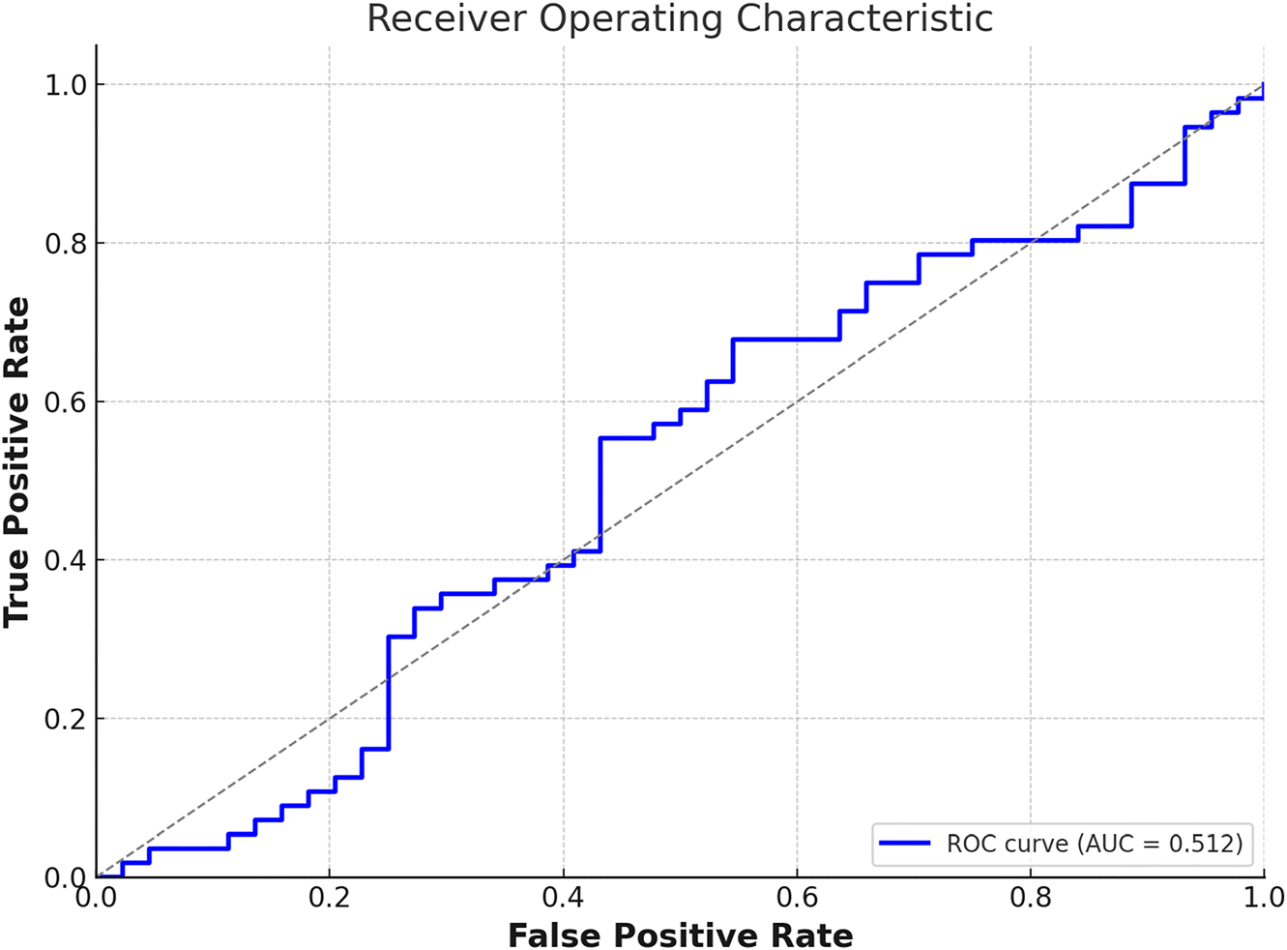
Receiver operating characteristic curve of the nomogram. Receiver operating characteristic (ROC) curve evaluating the discrimination performance of the nomogram; the area under the curve (AUC) was 0.512 (as shown on the plot).

**Figure 5:**
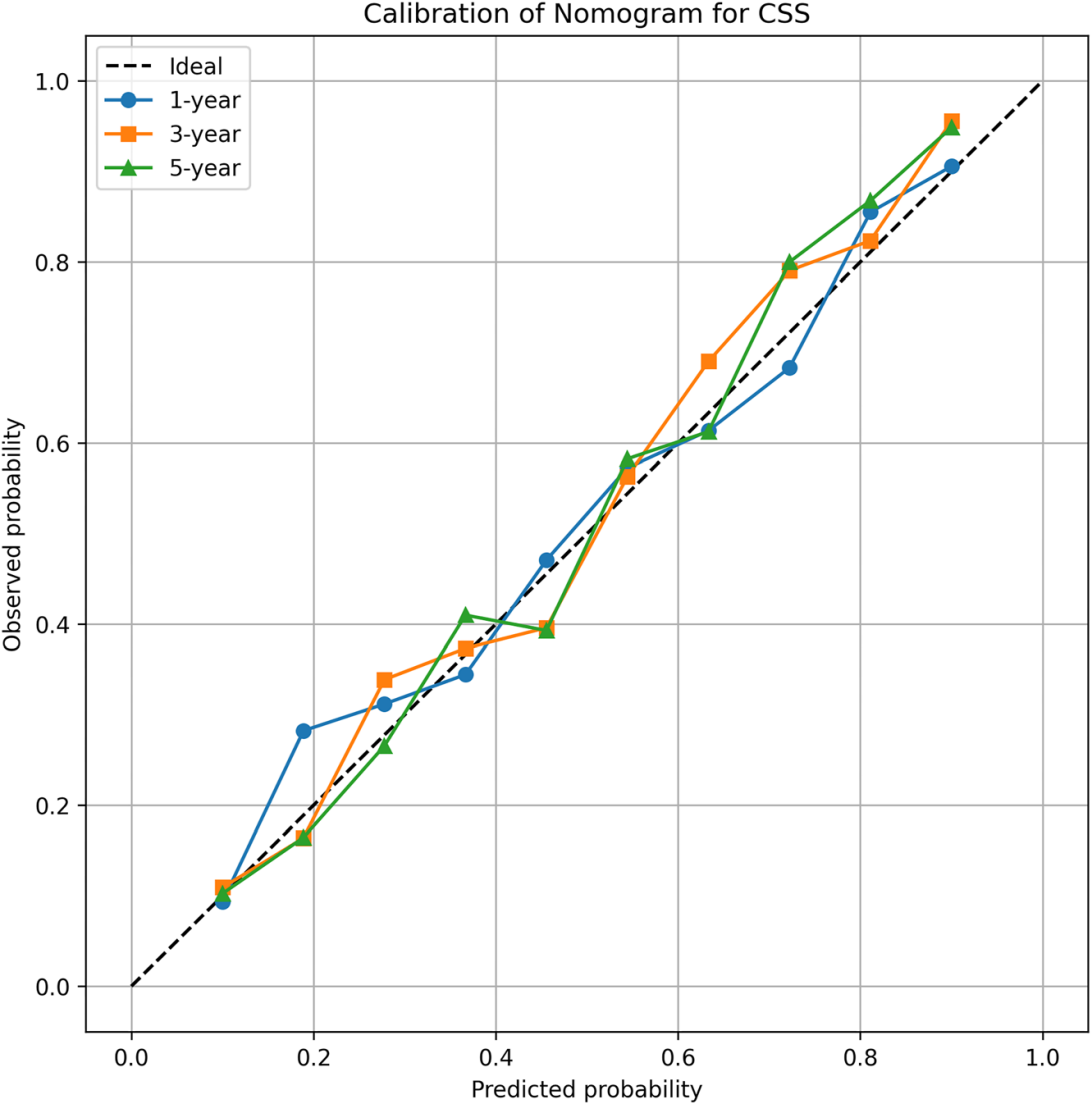
Calibration curves of the nomogram at 1, 3, and 5 years. Calibration plots comparing predicted versus observed 1-year (a), 3-year (b), and 5-year (c) cancer-specific survival probabilities. The diagonal line indicates perfect agreement between predictions and observations.

**Model discrimination:** Discrimination of the nomogram was evaluated using ROC analysis. The ROC curve is shown in Figure 4 and demonstrated limited discrimination, with an AUC of **0.512**, indicating near-random classification performance in this cohort. Calibration of the nomogram at 1, 3, and 5 years is shown in Figure 5. Accordingly, the nomogram should be interpreted as an exploratory prognostic tool requiring refinement and external validation in larger, independent cohorts before clinical application.

## 4 Discussion

Esophageal cancer is an aggressive malignancy with a poor prognosis, and improving progression-free survival remains a pressing clinical challenge. Growth factor signaling pathways involving receptors such as VEGFR2 and IGF-1R are increasingly being investigated for their prognostic and therapeutic roles in various cancers, including esophageal carcinoma [14], [15], [16]. Reliable prognostic biomarkers, if identified, could help guide treatment decisions and enhance risk stratification.

In the present study, two of the four receptors assessed – VEGFR2 and IGF-1R – were significantly associated with survival outcomes in patients with esophageal cancer. Specifically, high expression levels of VEGFR2 and IGF-1R correlated with shorter cancer-specific survival. In contrast, PDGFR and c-MET expression showed no significant association with survival (PDGFR: HR = 1.08, 95 % CI: 0.71–1.64; c-MET: HR = 1.12, 95 % CI: 0.74–1.69). The lack of association may be explained by functional redundancy among growth factor signaling pathways, a predominant role of VEGFR2/IGF-1R-driven mechanisms in this cohort, or limited statistical power due to the small number of events for these markers. Nevertheless, PDGFR and c-MET may still hold relevance as therapeutic targets or indicators of tumor biology, warranting further exploration in larger studies.

The biological functions of VEGFR2 and IGF-1R provide a rationale for their involvement in tumor angiogenesis and growth factor signaling [17], [18], [19], [20]. Recent evidence supports this view: VEGFR2 expression has been linked to microvessel density and tumor progression in esophageal cancer, and IGF-1R signaling is associated with chemoresistance and epithelial–mesenchymal transition in ESCC [21], 22]. Our findings thus add to the existing literature, suggesting that altered VEGFR2 signaling and angiogenic remodeling may contribute to adverse outcomes [17], and that IGF-1R influences key oncogenic processes – including proliferation, motility, and survival – through major pathways such as PI3K/AKT and Ras/MAPK [18], [19], [20].

Mechanistically, VEGFR2 is primarily expressed on tumor-associated endothelial cells and reflects angiogenesis rather than tumor cell proliferation per se. Low VEGFR2 may therefore indicate impaired tumor vasculature, leading to hypoxia-driven selection of more aggressive clones. Similarly, IGF-1R expression captures both tumor cell signaling and paracrine interactions within the microenvironment; low IGF-1R could signal loss of differentiation or adaptive pathway switching that favors invasive phenotypes. These interpretations are consistent with previous studies suggesting that activation or overexpression of VEGFR2- and IGF-1R-related signaling pathways may contribute to tumor progression and poor outcomes in solid tumors, including esophageal cancer [17], [18], [19], [20, 23].

Several factors may further explain this counterintuitive pattern. First, our IHC scoring does not cleanly separate tumor-cell versus endothelial expression, so “low VEGFR2” may serve as a surrogate for poor or dysfunctional tumor vasculature rather than reduced angiogenic drive. Second, aggressive tumors may downregulate canonical VEGFR2/IGF-1R signaling while compensatorily activating alternative pathways – so-called pathway switching – particularly under hypoxia-driven selection pressures that promote EMT-like phenotypes and clonal adaptation. Third, technical and analytical issues, including staining heterogeneity, inter-observer variability, and median dichotomization, can obscure dose-response relationships and misclassify biologically meaningful intermediate expression levels. Finally, residual confounding (e.g., treatment intensity, nutritional status, comorbidity burden) and the limited sample size cannot be ruled out. Thus, these findings should be viewed as hypothesis-generating and require independent validation.

Despite the paradoxical association, VEGFR2 and IGF-1R remain clinically relevant. Both pathways are mechanistically linked to angiogenesis, tumor growth, and therapeutic sensitivity or resistance in esophageal cancer and other solid tumors [17], [18], [19], [20, 23]. Their status may inform future strategies involving anti-angiogenic agents or IGF-1R-targeted therapies, though such approaches remain investigational.

Clinically, the nomogram integrating VEGFR2 and IGF-1R with TNM staging could aid in identifying patients at higher risk of recurrence or cancer-specific death, potentially guiding closer surveillance or adjuvant therapy selection. Similar integrative clinicomolecular nomograms have demonstrated improved prognostic accuracy over TNM staging alone in esophageal cancer and other gastrointestinal malignancies [24], 25]. Additionally, VEGFR2 and IGF-1R status could inform eligibility for targeted therapy strategies, such as VEGFR inhibitors or IGF-1R antagonists, and rational combinations with immune checkpoint inhibitors (ICIs), although clinical availability of these inhibitors in esophageal cancer remains limited and investigational [17], [18], [19], [20, 23]. Thus, while not yet standard-of-care, biomarker-guided therapy selection could be explored in prospective trials.

Clinically, our study reaffirmed that higher clinical stage, T stage, and N stage are strongly associated with worse prognosis as expected and align with the TNM classification system [26], 27]. Although TNM staging remains the principal tool to predict outcomes for esophageal cancer, it primarily reflects anatomical progression and may not fully capture biological heterogeneity [28], 29]. Recent endeavours have focused on prognostic prediction models combining molecular and clinical variables to enable more individualized survival predictions (eg, nomograms) [30]. Our findings support this approach, as the proposed nomogram integrates established staging variables with VEGFR2 and IGF-1R expression, consistent with earlier reports showing that combined clinical-molecular models may improve prognostic discrimination over TNM staging alone [25], 31], 32]. However, when evaluated by ROC analysis, the nomogram showed limited discrimination (AUC = 0.512), indicating performance near random classification in this cohort. This likely reflects the modest sample size, sparse events in certain clinicopathological strata, potential model instability, and the risk of overfitting given the number of predictors relative to events. Therefore, the model should be considered exploratory. It requires refinement – for instance, through alternative variable selection strategies, category collapsing or penalization methods, or different modeling approaches – and must undergo external validation in larger independent datasets before any clinical application.

Several factors may explain why high VEGFR2 and IGF-1R expression was associated with poor prognosis in this ESCC cohort. First, VEGFR2 overexpression may indicate increased angiogenic signaling and a more vascularized tumor microenvironment, which can support tumor growth and dissemination. Second, IGF-1R activation may enhance malignant phenotypes by promoting cell proliferation, survival, migration, and treatment resistance. Third, the combined elevation of angiogenic and growth factor signaling may reflect a biologically aggressive ESCC subtype. Nevertheless, because this was a retrospective single-center study with a modest sample size, these findings should be regarded as hypothesis-generating and require validation in larger cohorts.

Our results provide evidence that VEGFR2 and IGF-1R may function as independent prognostic biomarkers in esophageal cancer and may add to risk stratification when used alongside well-validated clinical parameters. Future studies should validate these findings in large, prospective, multicenter ESCC cohorts and further clarify the biological basis of the observed associations. Standardized molecular profiling and biomarker scoring in ESCC will be critical for advancing prognostic identification and ultimately enabling accurate, mechanism-informed therapeutic strategies. Also, larger multicenter cohorts are also needed to validate subtype-specific prognostic effects.

## 5 Conclusions

In conclusion, our findings suggest that higher IGF-1R and higher VEGFR2 expression are potential adverse prognostic markers for cancer-specific survival in patients undergoing curative-intent esophagectomy for ESCC. A clinicomolecular nomogram integrating these biomarkers with established clinicopathological factors was developed and internally evaluated; however, its discrimination was limited in this cohort (AUC = 0.512) and should be considered exploratory. Therefore, model refinement and external validation are required before clinical application, ideally in larger multicenter ESCC cohorts.

## Supplementary Material

Supplementary Material

Supplementary Material

Supplementary Material

Supplementary Material

Supplementary Material

Supplementary Material
