# Supplementary figures and images for: Growth factor receptors IGF-1R and VEGFR2 are associated with the prognosis of patients with esophageal cancer after esophagectomy
Source: Open Life Sci. 2026 Jul 27;21(1):20251354. doi: 10.1515/biol-2025-1354 (PMC13401192; doi:10.1515/biol-2025-1354)

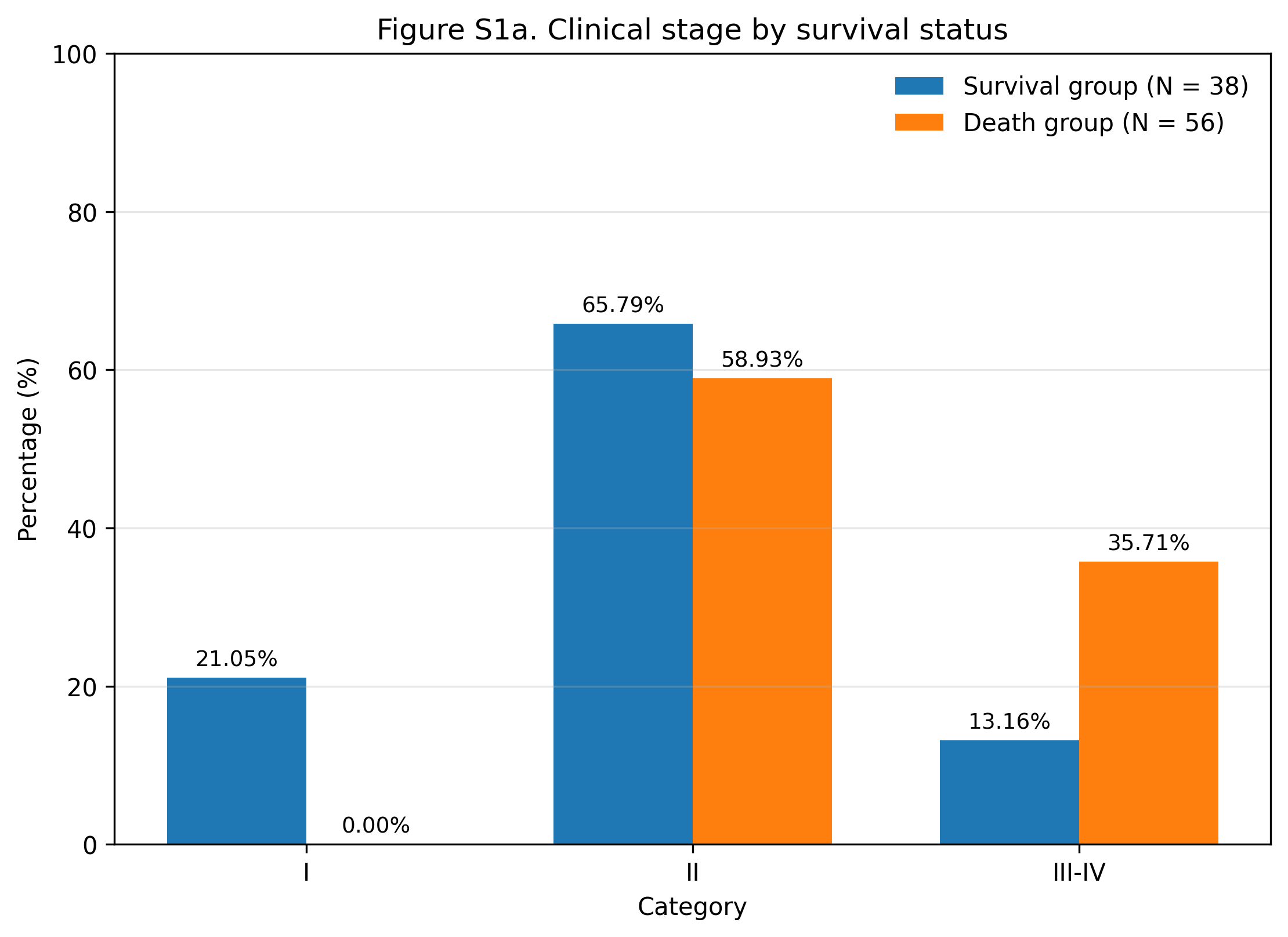

Supplement: Supplementary file 1 — Supplementary Material [file j_biol-2025-1354_suppl_001.png]

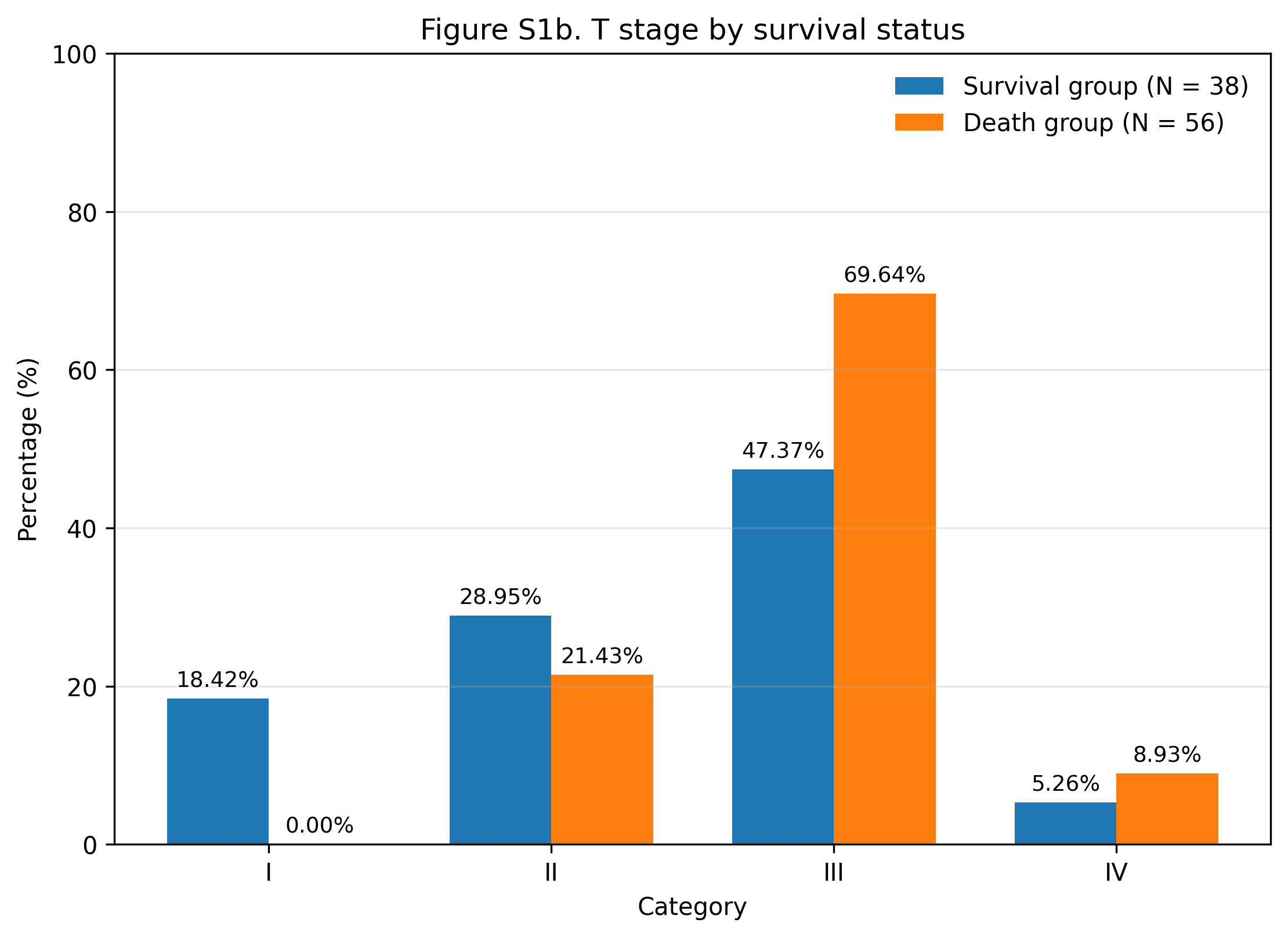

Supplement: Supplementary file 2 — Supplementary Material [file j_biol-2025-1354_suppl_002.png]

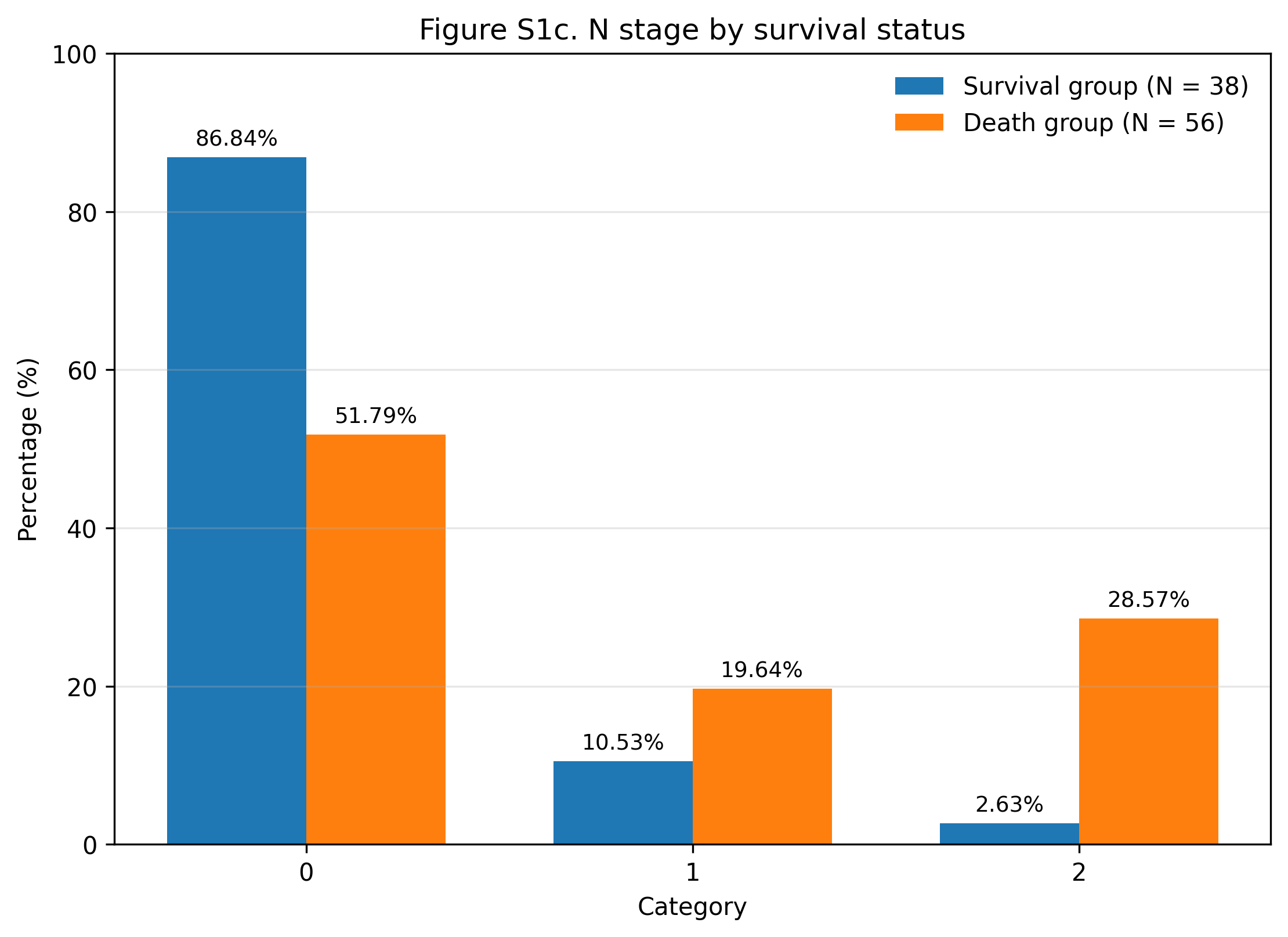

Supplement: Supplementary file 3 — Supplementary Material [file j_biol-2025-1354_suppl_003.png]

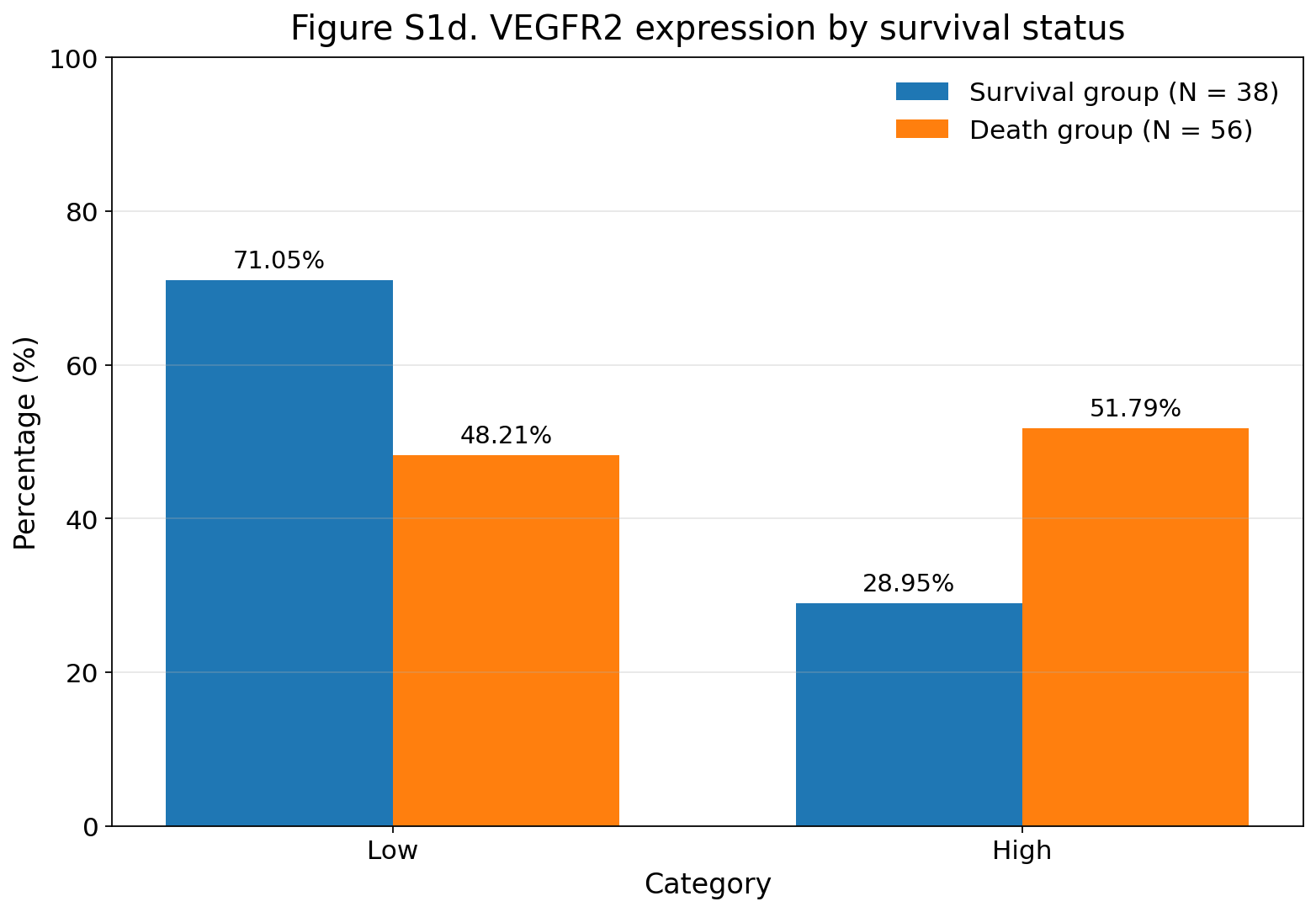

Supplement: Supplementary file 4 — Supplementary Material [file j_biol-2025-1354_suppl_004.png]

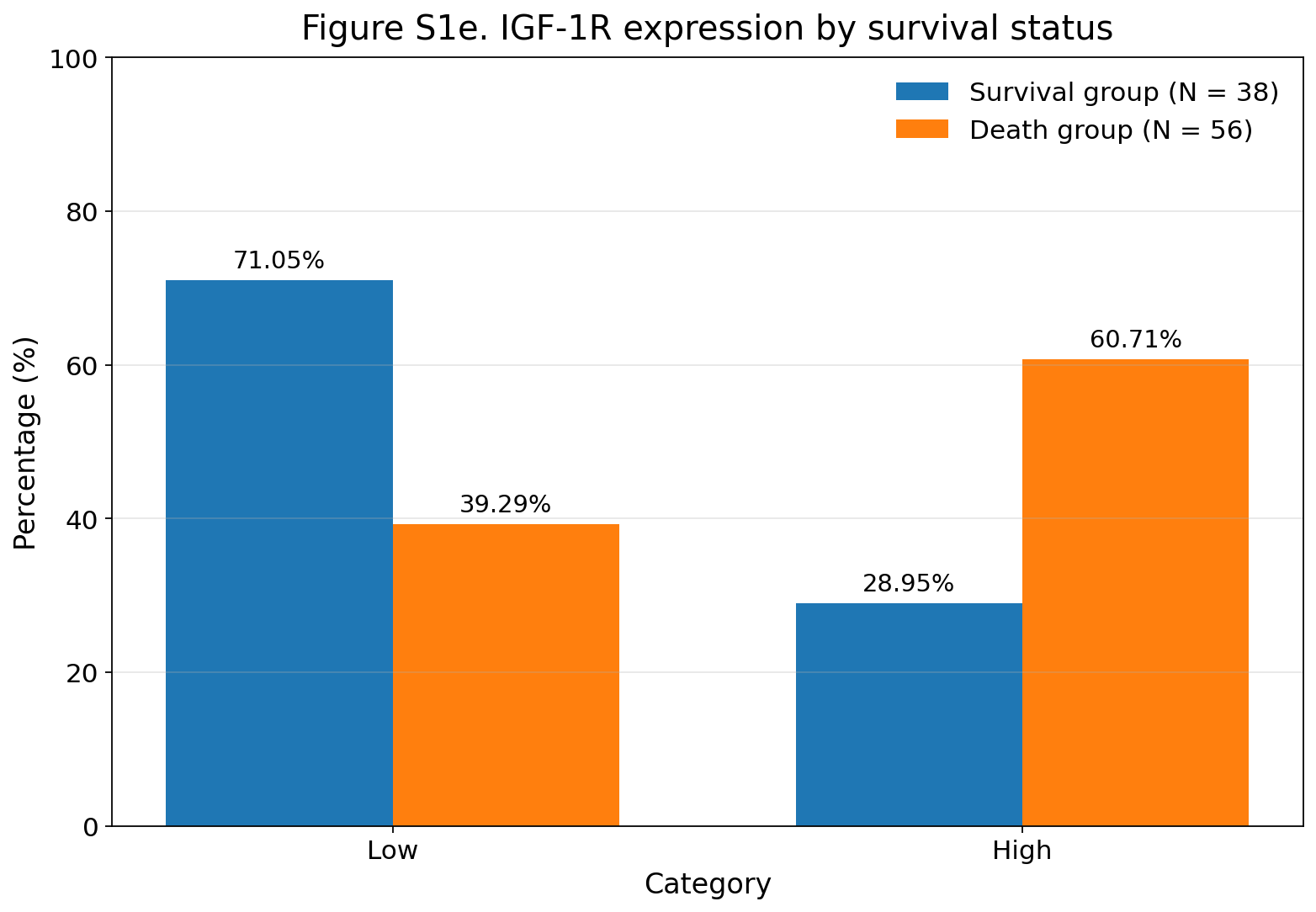

Supplement: Supplementary file 5 — Supplementary Material [file j_biol-2025-1354_suppl_005.png]
