# Supplementary material for: Growth factor receptors IGF-1R and VEGFR2 are associated with the prognosis of patients with esophageal cancer after esophagectomy
Source: Open Life Sci. 2026 Jul 27;21(1):20251354. doi: 10.1515/biol-2025-1354 (PMC13401192; doi:10.1515/biol-2025-1354)
Supplement: Supplementary file 6 — Supplementary Material [file j_biol-2025-1354_suppl_006.docx]

Figure S1. Comparison of key categorical clinical and molecular variables between survival and death groups in esophageal cancer patients.

(a) Clinical stage distribution: Percentage of patients in survival (N = 38) and death (N = 56) groups stratified by clinical stage (I, II, III–IV). Higher clinical stage is associated with increased mortality.

(b) T stage distribution: Percentage of patients in survival and death groups stratified by tumor invasion depth (T1–T4). Advanced T stage is associated with worse survival.

(c) N stage distribution: Percentage of patients in survival and death groups stratified by nodal involvement (N0–N2). Greater nodal involvement correlates with poorer outcomes.

(d) VEGFR2 expression: Percentage of patients with high or low VEGFR2 expression in survival and death groups. Higher VEGFR2 expression is associated with worse prognosis.

(e) IGF-1R expression: Percentage of patients with high or low IGF-1R expression in survival and death groups. Higher IGF-1R expression is associated with worse prognosis.
